# Beyond generalized anxiety: the association of anxiety sensitivity with disordered eating

**DOI:** 10.1186/s40337-023-00890-0

**Published:** 2023-10-02

**Authors:** Maria Bazo Perez, Timothy B. Hayes, Leslie D. Frazier

**Affiliations:** Department of Psychology, Florida International University, 11200 SW 8th Street, Miami, FL 33199 USA

**Keywords:** Anxiety sensitivity, Generalized anxiety, Disordered eating, Structural equation modeling, Eating Attitudes Test-26, Anxiety Sensitivity Index-3, Physical concerns, Cognitive concerns, Social concerns

## Abstract

**Background:**

Anxiety and eating disorders (EDs) are rising at alarming rates. These mental health disorders are often comorbid, yet the factors associated with their comorbidity are not well understood. The present study examined a theoretical model of the pathways and relative associations of anxiety sensitivity (AS) with different dimensions of ED risk, controlling for generalized anxiety.

**Methods:**

Participants (*N* = 795) were undergraduate students with an average age of 21 (*SD* = 4.02), predominantly female (71%), and Hispanic (71.8%). Participants completed an online survey with established measures of AS (i.e., Anxiety Sensitivity Index-3; ASI-3), general anxiety (i.e., Beck Anxiety Inventory; BAI), and eating behaviors (i.e., Eating Attitudes Test-26; EAT-26).

**Results:**

The results of our structural equation models indicated that AS subscales were significantly associated with dimensions of the EAT-26, even when controlling for generalized anxiety. Specifically, the ASI-3 factors reflecting cognitive and social concerns provided the most consistent significant associations with EDs. Whereas reporting higher cognitive concerns was associated with higher ED symptoms (e.g., reporting the urge to vomit after a meal), reporting higher social concerns was associated with fewer ED symptoms. These differential results may suggest risk and resilience pathways and potential protective or buffering effects of social concerns on ED risk.

**Discussion:**

Findings advance understanding of the role of AS in the comorbidity of anxiety and EDs, demonstrating the strong association of AS with ED pathology. These findings provide cognitive indicators for transdiagnostic therapeutic intervention in order to reduce the risk of EDs.

**Supplementary Information:**

The online version contains supplementary material available at 10.1186/s40337-023-00890-0.

## Abstract (plain language summary)

Many people with anxiety disorders also have high rates of eating pathology, and vice versa. Teasing apart the factors that may contribute to this co-morbidity can provide important information for psychotherapeutic prevention and intervention. In this study we examine the contributions of anxiety sensitivity, also known as the ‘fear of fear,’ beyond that of generalized anxiety in its associations with eating disorder outcomes. Our findings show that the cognitive concerns of anxiety (i.e., thinking about being anxious) are associated with higher urges to purge after eating. Higher social concerns with anxiety (i.e., concerns that others will know one is anxious) related to fewer symptoms of eating disorders. Taken together our findings illuminate that considering anxiety sensitivity may be helpful for the diagnosis and treatment of eating disorders.

**Supplementary Information:**

The online version contains supplementary material available at 10.1186/s40337-023-00890-0.

## Introduction

Eating disorders (EDs) represent a serious and growing health concern affecting 9% of the population worldwide [1]. Over the last two decades, the prevalence of EDs has risen from 3.5 to 7.8% [1]. Moreover, EDs are associated with high rates of psychiatric comorbidity [2–4], with numerous studies demonstrating the comorbidity between anxiety and eating pathology [5–11]. This is concerning, given that the lifetime prevalence of anxiety is approximately 33% [12] and also rising [13]. Even more concerning is the estimate that 80% of EDs go undiagnosed, and 75% of individuals who have symptoms do not seek treatment [14, 15]. Therefore, it is important to understand the associations between anxiety and eating disorder risk, especially in non-clinically diagnosed populations. In the present study, we focus on how anxiety sensitivity (AS)–the fear of fear–might relate to eating disorder risk above and beyond more commonly studied measures of general anxiety.

### Comorbidity among anxiety and eating disorders

Hudson and colleagues [3] analyzed the National Comorbidity Survey Replication (NCS-R; [16], a population-based sample, finding that over 50% of individuals diagnosed with EDs also reported a diagnosis of anxiety disorder.^1^ In individuals with EDs, anxiety was associated with binge eating [17, 18], vomiting [17, 18], and caloric restriction [19]. This association has also been established across samples with sub-clinical EDs and weight concerns [20, 21]. Further, high comorbidity has been consistently found [22] across different age groups, from adolescence [20, 22–24] to adulthood [8], including emerging adults [25].

Generalized anxiety disorder (GAD) is most commonly comorbid in individuals with EDs [9, 26]. The core feature of GAD, worry, is often elevated in individuals with EDs when compared with a non-clinical sample, and may play a role in eating symptomatology, representing a construct that is elevated in both diagnoses [27]. Specifically, worry is associated with food and weight [28, 29], eating pathology (e.g., fear of weight gain, inability to recover, engaging in ED behaviors like vomiting or exercise [30]; and may negatively impact the course of eating pathology relating to poorer treatment outcomes [9, 31]. Individuals with EDs who report comorbid anxiety problems, also report that symptoms of anxiety preceded the development of eating pathology in 60–90% of cases [32–35]. However, anxiety disorders do not always precede eating pathology [32], and some researchers have argued that EDs may exacerbate anxiety symptomology [9]. Nevertheless, anxiety is clearly one pathway in the genesis of eating pathology [33]. Although the mechanisms by which anxiety impacts eating pathology are still unclear [32], the high prevalence of comorbidity demands further research to identify transdiagnostic, coactive processes, and points of intervention. Moreover, approaching EDs from a generalized anxiety perspective may not be the most effective at illuminating underlying mechanisms, as there are different aspects of anxiety that may affect behavior differently, and therefore, we examine AS.

### Anxiety sensitivity and eating disorders

Anxiety sensitivity is related to but distinct from trait anxiety (or the tendency to experience anxiety across many situations and experiences) [34]. A cognitive construct, AS is the belief that the physical sensations that accompany anxiety will lead to ‘catastrophic outcomes’ such as dangerous physical symptoms or social embarrassment [35, 36]. It is the feeling of dread associated with anxiety-related bodily sensations (e.g., racing heart, butterflies in the stomach, quivering hands, and feelings of loss of control over bodily sensations [37–39]. Thus, AS is the fear of somatic arousal [40–42]. Individuals who are high in AS tend to amplify and misinterpret bodily sensations and symptoms of anxiety [37, 43, 44]. In moments of emotional distress, individuals may engage in maladaptive short-term affect regulation, and AS has been associated with heightened sensitivity to negative affect [45–48].

High levels of negative affect, related to AS, are a shared vulnerability in the emergence and maintenance of EDs and other internalizing disorders [49–52]. Individuals who report disordered eating also have higher levels of AS [53], and tend to overeat in response to negative emotions [54]. Higher levels of AS may lead to binge eating as a means to reduce emotional distress [55]. When levels of AS are high, it may potentiate the aversiveness of negative affective or somatic states [45, 46, 56]. Due to the heightened sensitivity of those negative emotions, individuals with AS may be more likely to engage in behavioral efforts to reduce their distress through maladaptive and pathological eating [56, 57]. Hearon and colleagues [40] found through ecological momentary assessment, that individuals with high levels of AS engaged in eating behaviors (i.e., calorie consumption) followed by high levels of negative affect.

The Anxiety Sensitivity Index-3 (ASI-3) [58] is an extensively used multidimensional measure of AS. The first dimension, cognitive concerns, refers to the fear of being mentally unable to control cognitions, the second one, physical concerns, focuses on the fear of experiencing the physiological symptoms related to anxiety, and the third dimension, social concerns, refers to the fear of one’s symptoms being publicly observable [58]. Little research to date has examined how the different dimensions of AS, (i.e., physical, social, and cognitive concerns) may differently predict EDs. One study showed that the cognitive concerns dimension of the AS, as measured by the ASI-3 [58] was significantly associated with disordered eating measured by the Eating Attitudes Test-26 (EAT-26) [59], suggesting that individuals high in this dimension may engage in maladaptive eating behaviors as a means to reduce the unwanted internal states, such as thoughts, emotions or physical symptoms [53]. In individuals with a clinically diagnosed ED, all three dimensions of the ASI-3 [60] were positively correlated with the severity of ED symptomology. However, while controlling for comorbid psychopathology, only social and physical AS were related to ED symptoms. In that study, higher levels of social AS were related to elevated ED symptom severity, whereas higher physical AS was unexpectedly related to lower ED symptom severity. The authors [61] suggest that the negative association of physical AS on ED severity was a statistical suppression effect. Taken together, there is a clear association among AS and EDs. Understanding the differential pathways of association among dimensions of AS on ED outcomes is important because the experience of AS may relate to the heterogenous symptomology of EDs [62].

### The present study

**Fig. 1:**
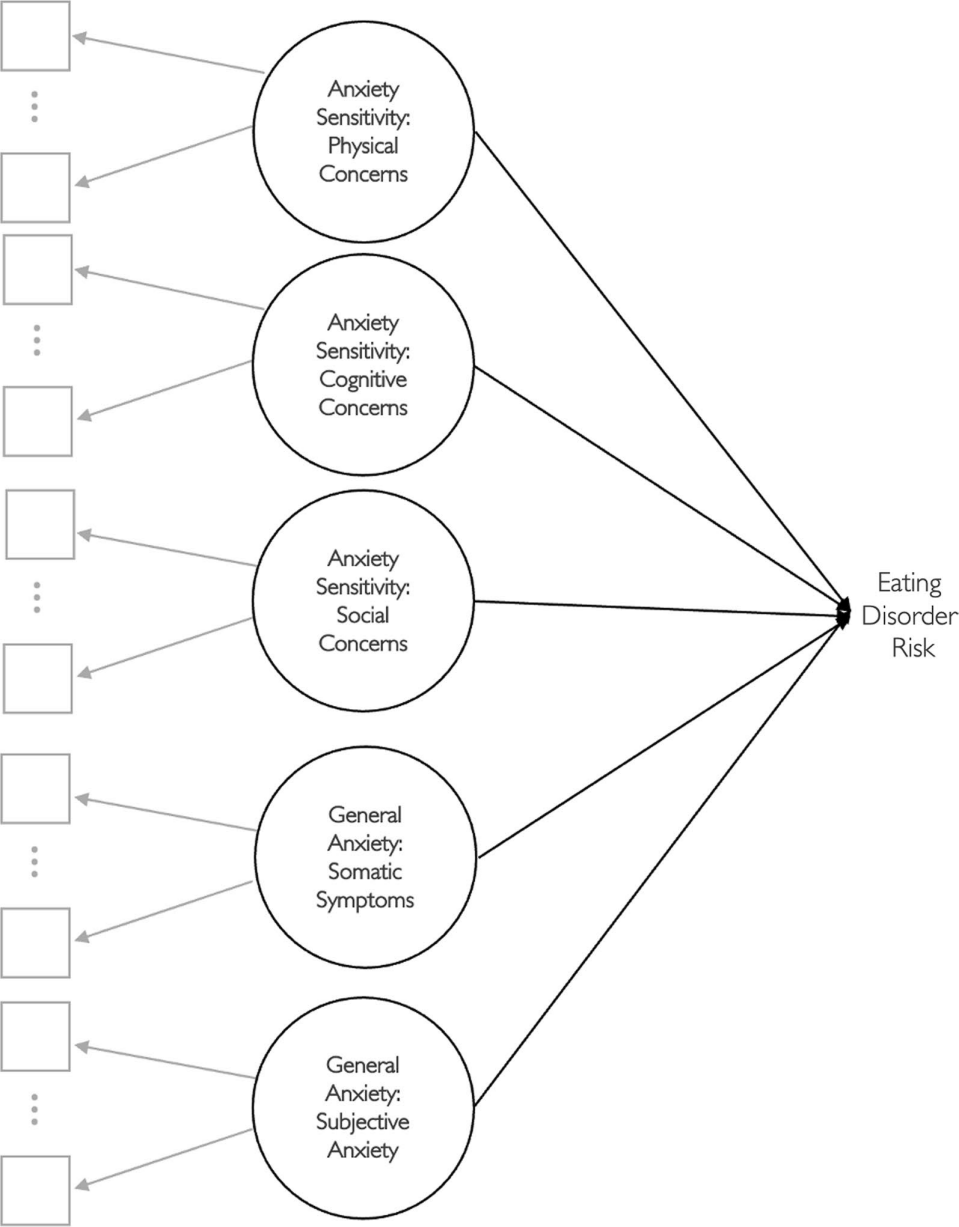
General, conceptual path diagram of the latent variable regression approach to incremental validity testing relating the anxiety sensitivity and general anxiety dimensions to eating disorder risk. *Note:* For ease of reading, the diagram omits exogenous variances and covariances and the endogenous disturbance. Ellipses, $\vdots$, between factor model indicators suggest that additional indicators may load on each factor model (but are omitted to conserve space). Solid black vs solid grayscale lines are used to visually distinguish the regression model from the measurement models, respectively. Solid black one-headed arrows indicate regression paths

The rationale for this study was to go beyond prior research that has established associations between general anxiety and AS and EDs, by examining their unique contributions within the same model. Specifically, in this study, we examine the linkages among the three subscales of the ASI-3 [38] and their differential associations with four factors of disordered eating, after controlling for the associations with generalized anxiety. Broadly, if AS remains associated with eating disorder risk even after partialling out overlapping variance shared with general anxiety, this would support the idea that AS is a distinct and separable construct exhibiting potentially important incremental utility [63] in the eating disorder domain. We expected a-priori that the three AS constructs (i.e., physical, cognitive, and social concerns) will be associated with the EAT-26 above and beyond general anxiety. Figure 1 shows a general conceptual diagram of the anticipated regression of disordered eating on AS and generalized anxiety.

## Method

### Participants

The sample comprised 795 undergraduate students, predominantly female (71%), with an average age of 21 (*SD* = 4.02), who were recruited through the Psychology department participation pool and volunteered to take part in an online survey in exchange for course credit for participation. Inclusion/exclusion criteria were: (1) over aged 18, (2) able to read English. Self-reported race/ethnicity: Hispanic (61.6%), African American (9.6%), White Non-Hispanic (7.4%), South Asian (e.g., Indian, Pakistani, 1.1%), Asian/Asian American (1.4%), Native American (0.1%), Other (4.9%), and no response (14%). Participants (73%) reported living with family and over half of the sample (51.5%) indicated an annual household income of less than $50,000. The sample was consistent with the characteristics of the major urban public research university and the surrounding community it serves. Physical characteristics reported by participants included height, range from 144.78 to 195.58 cm (*M* = 166.17 cm, *SD* = 9.42 cm), and weight, range from 39.01 to 139.71 kg (*M* = 67.87 kg, *SD* = 15.98 kg). Reported values were used to compute participants’ body mass index (BMI; *M* = 24.96, *SD* = 5.13), which on average represented the ceiling of normal BMI.

### Measures

#### Anxiety Sensitivity Index-3

Anxiety sensitivity was measured using the ASI-3 [58]; an 18-item version of the original ASI [38] assessing participants’ concerns regarding arousal-related sensations across three dimensions with six items each: (1) cognitive concerns, (2) physical concerns, and (3) social concerns. Likert-type response scales ranged from 0 (*very little*) to 4 (*very much*). The total scale ranges from 0 to 72, and higher scores are indicative of higher sensitivity to arousal sensations. In the present sample, the average total score was 22.22 (*SD* = 16.65). Internal reliability was α = 0.89 for cognitive concerns, α = 0.84 for physical concerns, and α = 0.92 for social concerns.

#### Beck Anxiety Inventory

Anxiety was measured using the Beck Anxiety Inventory (BAI) [64], a 21-item scale designed to assess generalized symptoms of anxiety. It is comprised of two subscales: (1) somatic symptoms, and (2) subjective anxiety [68]. Participants indicated how much they have been bothered in the past month by symptoms of anxiety on a 4-point Likert scale from 0 (*not at all)*, to 3 (*severely—it bothered me a lot*). Total scores range from a low of 0 to a high of 63. Scores on the BAI are classified as minimal anxiety (0–7), mild anxiety (8–15), moderate anxiety (16–25), and severe anxiety (30–63). In the current sample, the average total score was 38.53 (*SD* = 14.99). Internal reliability was α = 0.93 for somatic symptoms subscale and α = 0.90 for subjective anxiety subscale.

#### Eating Attitudes Test-26

The EAT-26 [59] was used to assess symptoms of disordered eating and ED symptomology in both non-clinical and clinical populations. An effective screening tool, it has a reported sensitivity of 90% when measured against clinical diagnostic interviewed based on DSM-IV criteria [65]. However, some more recent studies have found insufficient to moderate sensitivity of the EAT-26 to detect full or partial EDs [66]. The original structure of the EAT-26 consists of three subscales: (1) dieting, (2) bulimia and food preoccupation, and (3) oral control. Participants responded on a 6-point Likert scale with answer choices ranging from 1 (*never*) to 6 (*always*). Following Garner et al., [59], answers 1 through 25 are recoded for frequency to represent: 0 (*never, rarely or sometimes)*, 1 (*often)*, 2 (*usually)*, and 3 (*always),* and question 26 is recoded using the reverse, such that higher scores indicate greater endorsement of disordered eating behaviors on each subscale. In the present sample, the average total score was 9.68 (*SD* = 9.15), close to the cutoff of 11 and above for risk of overweight and bulimic and binge-purge symptoms established in recent research [66–68].

Despite its extensive use, there is an emerging body of literature questioning the factor structure of the EAT-26, as the three-factor structure originally developed in an AN female sample does not perform similarly in non-clinical and non-SWAG (Skinny White Affluent Girls) stereotyped populations [69–72]. Different factorial structures have been reported in non-clinical samples [71], different cultures and ethnic groups [66, 70, 72–74], and different genders [75]. Therefore, in the present study, we analyzed the factorial structure of the EAT-26 at the item and subscale level in order to evaluate how this measure performed in our mostly Hispanic and female sample. Internal reliability for the total score was α = 0.87.

### Procedure

These data are part of a large cross-sectional study on weight- and health-related concerns in college students conducted at a large public, urban university in the southeastern United States. Upon signing up, participants received an anonymous Qualtrics survey link. Those who provided informed consent continued on to complete the survey.

## Results

In line with recommendations from the methodological literature [63, 76], we tested our incremental validity hypotheses about the associations of AS with disordered eating, above and beyond general anxiety, using a latent variable structural equation modeling (SEM) approach that separated true score variation in each construct from measurement error. We fit all SEM models using the lavaan package in R [77, 78] and Mplus version 8 [79].

### Assessing the factor structure of all scales

#### Approach to factor model estimation and handling of missing data

Before running our full latent variable regression model (depicted conceptually in Fig. 1), we ran a series of confirmatory factor analysis (*CFA*) models assessing the fit of each measurement model in our sample. When reviewing the original scale validation study for the ASI-3, we noted that Taylor et al. [80] conducted their CFA models using categorical factor analysis methods employing weighted least squares estimation procedures based on polychoric correlations–a method known in the quantitative literature for being appropriate to handle ordinal outcomes with five or fewer categories [81, 82]. Because the items of the BAI and EAT-26 are assessed using four-point ordinal response scales,^2^ we opted to apply ordinal factor analysis methods to these items as well.

Thus, following Taylor et al. [80] example, for our confirmatory and exploratory factor analyses, we used WLSMV (weighted least squares mean and variance adjusted) estimation which allowed us to treat the scaling of the categorical items appropriately while also obtaining the standard battery of SEM model fit indices [79, 83]. Because this approach to estimation requires complete data, we initially hoped to use categorical multiple imputation methods to fill in the missing values, allowing us to easily apply complete data WLSMV methods to each imputed dataset in a subsequent step. Unfortunately, when we attempted multiply impute all item-level data using categorical imputation methods in the Blimp software package [84], the imputation algorithm failed to reach convergence despite increasing the number of Bayesian MCMC iterations to extremely large values (in the hundreds of thousands and even the millions), likely due to the large number of ordinal items to be imputed.

Failing our first-choice multiple imputation strategy, we adopted a two-pronged approach to missing data handling. First, we estimated all of our primary models using WLSMV estimation on the *N* = 697 cases with complete data, deleting the 12.3% of cases with missing values. This approach allowed us to assess the model fit associated with all initial confirmatory factor analysis models. Once this was accomplished, we then proceeded to verify our final structural regression model results in a second step by rerunning the model with Bayesian missing data estimation procedures in Mplus, using the estimates from our final WLSMV model as starting values to aid model estimation and help ensure convergence. We chose a Bayesian approach to model estimation under missing data because it is known to be faster and more computationally tractable than frequentist estimation using FIML for categorical indicators (a procedure that requires computationally costly numerical integration methods in Mplus and tends to break down as the number of latent variables increases beyond 2 or 3) [79]. The pairwise proportion of complete cases on each pair of variables obtained from the Bayesian analysis of our final model in Mplus ranged from 0.957 to 1.00.

#### Assessing the fit of the ASI and BAI and reevaluating the fit of the EAT-26

Fit statistics for all factor models are displayed in Table 1. The 3-factor structure of the ASI-3 [58] and the 2-factor structure of the BAI [64] exhibited generally acceptable fit [85].^3^ By contrast, the original three-factor structure of the EAT-26 Garner et al. [59] showed unacceptable model fit across nearly all metrics. This was not surprising, given recent criticisms of the EAT-26 in the literature [86–88]. Therefore, we conducted the EFA in Mplus, using WLSMV estimation to handle the categorical indicators in the same manner as our CFA. In this analysis, we extracted estimates and fit indices for 1- through 7-factor models.

**Table 1:** Model fit indices

	Anxiety Sensitivity Inventory-3	Beck Anxiety Inventory	EAT-26 Original Structure	EAT-26 4-factor EFA	EAT-26 4-factor CFA	Latent Variable Regression (Full Model)
Chi-square	645.37	1703.98	1547.93	373.5	132.13	2743.48
* df*	132	188	296	227	38	1132
* p*	< .001	< .001	< .001	< .001	< .001	< .001
CFI	0.98	0.95	0.85	0.98	0.97	0.96
TLI	0.97	0.94	0.83	0.98	0.96	0.96
RMSEA	0.07	0.10	0.07	0.03	0.06	0.04
90% CI	[0.07, 0.08]	[0.1, 0.11]	[0.07, 0.08]	[0.02, 0.03]	[0.05, 0.07]	[0.04, 0.05]
* p*_*close*_	< .001	< .001	< .001	1.000	.156	1.000
SRMR	0.04	0.07	0.14	0.05	0.09	0.06

All models were fit using WLSMV estimation in lavaan, except for the EFA model in main table column 4, which was conducted using WLSMV estimation in Mplus. Fit statistics from lavaan are reported are from the robust column of output

To arrive at a final factor structure for the EAT-26 items, we used a combination of statistical and substantive criteria. Model fit was subpar for the 1- and 2-factor models so they were ruled out. Next, because a latent variable requires at least two indicators to be identified in a larger model featuring two or more latent variables [88–91], we only considered models in which at least two indicators exhibited high standardized factor loadings (≥ 0.7) on every factor.^4^ This criterion further ruled out the 5-, 6- and 7-factor models from consideration. To determine among the 3- and 4-factor models, we only considered factor loadings ≥ 0.3, and we dropped from consideration any indicator that did not load highly on any factor with a standardized loading ≥ 0.7. Finally, because we wanted to impose *perfect simple structure* in the measurement models in our final SEM [93, 94], we dropped any indicator featuring a cross-loading ≥ 0.3.

The factor structure of the 3- and 4-factors models were largely overlapping. The difference was that the fourth factor extracted in the larger model included the two purging items from the EAT-26. Because these items were substantively meaningful indicators of ED risk, we opted to retain the 4-factor structure as our final model. Table 2 shows the standardized loadings of all items retained in our final model. All factors in this model, except for factor 3, “preoccupation with thinness,” feature either two or three indicators. On factor 3, the three highest loading indicators–items 1, 11, and 14–all clearly grouped around a common substantive meaning. Because the remaining two items (10 and 12) were not as closely aligned in their substantive meaning and because a latent factor in an SEM only requires 3 indicators to be identified (not 5), we opted to drop these items from our subsequent analyses.

**Table 2:** Standardized loadings from 4-factor EFA and CFA analyses of EAT-26 items

	EFA				CFA			
	Diet/ carb restriction	Pressure to eat	Preoccupation with thinness	Purging	Diet/ carb restriction	Pressure to eat	Preoccupation with thinness	Purging
7. Particularly avoid food with a high carbohydrate content (i.e. bread, rice, potatoes, etc.)	**0.77**	− 0.01	0.16	0.03	**0.88**	0	0	0
16. Avoid foods with sugar in them	**0.79**	0.04	− 0.05	0.01	**0.72**	0	0	0
17. Eat diet foods	**0.73**	0.03	0.25	− 0.01	**0.80**	0	0	0
8. Feel that others would prefer if I ate more	0.02	**0.85**	0.02	− 0.07	0	**0.83**	0	0
13. Other people think that I am too thin	− 0.10	**0.90**	− 0.10	0.07	0	**0.87**	0	0
20. Feel that others pressure me to eat	− 0.03	**0.81**	0.26	− 0.03	0	**0.84**	0	0
1. Am terrified about being overweight	0.03	− 0.03	**0.89**	− 0.14	0	0	**0.86**	0
11. Am preoccupied with a desire to be thinner	0.01	− 0.03	**0.91**	0.05	0	0	**0.91**	0
14. Am preoccupied with the thought of having fat on my body	− 0.03	0.10	**0.93**	− 0.10	0	0	**0.90**	0
10. Feel extremely guilty after eating	− 0.08	0.07	**0.79**	0.22	–	–	–	–
12. Think about burning up calories when I exercise	0.23	− 0.17	**0.73**	− 0.01	–	–	–	–
9. Vomit after I have eaten	0.08	0.15	− 0.05	**0.83**	0	0	0	**0.78**
25. Have the impulse to vomit after meals	0.17	0.21	− 0.01	**0.82**	0	0	0	**1.07**

Item wordings are reworded with electronic permission from the original scale by Garner et al. (1987). The remaining EAT-26 items not shown here were included in the EFA analysis, but ultimately excluded from the final factor structure based on the decision-making criteria described in the main manuscript. High standardized factor loadings (≥ .7) are presented in bold text

It is worth noting the correspondence between the factor structure we uncovered and those found in the previous literature. After dropping items 10 and 12 from the preoccupation factor, the structure of this factor was identical to the three-indicator ‘self-perception of body shape’ factor identified by Ocker et al. [86] and also mirrored the highest loading items from factor 1 in the supplemental materials of Maïno et al. [87]. The other factors identified in our categorical EFA exhibited similarities with previous work as well: the three indicators in our “diet/carb restriction” factor are also featured in Ocker et al. [86] “dieting” factor and Maïno et al. [87] factor 4. Similarly, the items in our “pressure to eat” and “purging” factors were the same indicators that exhibited the highest loadings in Maïno et al. [87] factors 2 and 3, respectively. Although we conducted our categorical EFA analyses prior to discovering and reviewing this prior work^5^–such that the decision-making procedure described here was not influenced by the results of these previous studies–the similarity of our results to those of Ocker et al. [86] and Maïno et al. [87] is broadly encouraging.

Following our EFA analysis, we conducted a CFA of our four-factor solution to assess the fit of this model to our data. We emphasize that the purpose of this CFA was *not* to ‘confirm’ our exploratory model in the same (training) dataset^6^ but was, rather, to establish that a CFA approach, imposing perfect simple structure by allowing items to load on one and only one factor (thereby imposing zero loading constraints in the remaining entries of the factor loading matrix), would exhibit acceptable fit to our data. As seen in Table 1, the model fit was high in both the 4-factor EFA and the 4-factor CFA. Encouraged by these results, we proceeded to our SEM analyses.

### Approach to latent variable regression model specification

**Fig. 2:**
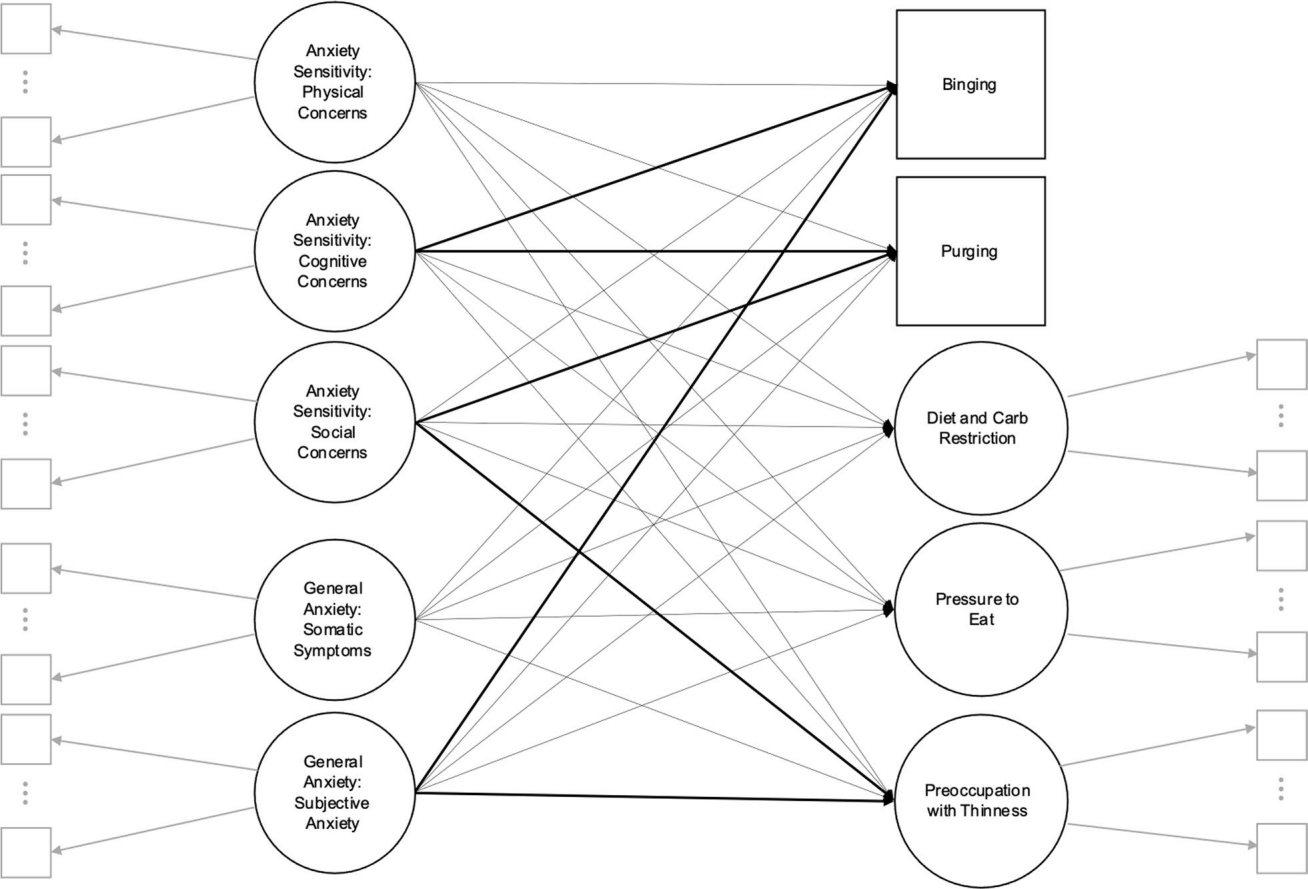
Path diagram of the final latent variable regression model. *Note:* For ease of reading, these diagrams omit: **a** exogenous variances and covariances as well, **b** item residuals (implied unique factors), **c** endogenous disturbances, and **d** disturbance variances and covariances. Once again, ellipses, $\vdots$, between factor model indicators suggest that additional indicators may load on each factor model (but are omitted to conserve space). And once again, solid black vs solid grayscale lines are used to visually distinguish the regression models from the measurement models, respectively. One-headed arrows represent regression relationships. Bolded lines represent paths that reached significance in our final analyses (see Model 3 in Table 3)

Figure 2 displays our full latent variable regression^7^ model.^8^ Note that we added the single EAT-26 item 4 assessing binging behaviors as a model outcome, based on the substantive importance of this type of ED behavior. Although utilizing a single item for this construct is not ideal, this approach allowed us to assess binging behaviors rather than omitting this construct from the model or attempting to force this binging item to hang together with other scale items that do not share the same meaning (that is, that do not ask about binging behavior directly).

**Table 3:** Standardized latent regression results of EAT-26 outcomes regressed on general anxiety and anxiety sensitivity

	Model 1: General Anxiety Only					Model 2: Anxiety Sensitivity Only					Model 3: Full Model				
	*Est*	*sr*^2^	*SE*	*z*	*p*	*Est*	*sr*^2^	*SE*	*z*	*p*	*Est*	*sr*^2^	*SE*	*z*	*p*
Binging regressed on:															
Physical concerns						0.11	.004	0.12	0.94	.346	0.07	.001	0.12	0.57	.566
Cognitive concerns						**0.46**	**.053**	**0.12**	**3.92**	** < .001**	**0.42**	**.042**	**0.12**	**3.51**	** < .001**
Social concerns						− 0.27	.019	0.15	− 1.86	.063	** − 0.33**	**.027**	**0.15**	** − 2.15**	**.032**
Somatic symptoms	− 0.36	.018	0.25	− 1.45	.148						− 0.40	.023	0.25	− 1.65	.100
Subjective anxiety	**0.63**	**.057**	**0.24**	**2.63**	**.009**						**0.57**	**.043**	**0.25**	**2.26**	**.024**
* R*^2^	.11					.12					.17				
Purging regressed on:															
Physical concerns						0.18	.011	0.16	1.11	.267	0.20	.012	0.17	1.14	.252
Cognitive concerns						**0.64**	**.102**	**0.13**	**4.90**	** < .001**	**0.66**	**.105**	**0.13**	**4.92**	** < .001**
Social concerns						** − 0.56**	**.081**	**0.20**	** − 2.88**	**.004**	** − 0.57**	**.079**	**0.22**	** − 2.58**	**.010**
Somatic symptoms	− 0.46	.030	0.38	− 1.22	.222						− 0.54	.042	0.36	− 1.52	.127
Subjective anxiety	0.59	.050	0.37	1.59	.111						0.48	.030	0.43	1.11	.269
* R*^2^	.06					.18					.22				
Diet/carb restriction regressed on:															
Physical concerns						− 0.06	.001	0.12	− 0.51	.607	− 0.07	.001	0.13	− 0.52	.606
Cognitive concerns						0.08	.002	0.13	0.58	.560	0.08	.001	0.14	0.56	.577
Social concerns						− 0.06	.001	0.13	− 0.49	.627	− 0.07	.001	0.13	− 0.52	.606
Somatic symptoms	− 0.09	.001	0.2	− 0.42	.671						− 0.08	.001	0.21	− 0.39	.697
Subjective anxiety	0.06	.000	0.2	0.29	.771						0.09	.001	0.21	0.42	.678
* R*^2^	.00					.00					.01				
Pressure to eat regressed on:															
Physical concerns						− 0.29	.026	0.15	− 1.91	.056	− 0.31	.030	0.16	− 2.00	.045
Cognitive concerns						0.28	.020	0.17	1.64	.101	0.25	.015	0.17	1.48	.139
Social concerns						0.16	.007	0.15	1.10	.270	0.13	.004	0.15	0.90	.370
Somatic symptoms	− 0.06	.000	0.22	− 0.25	.803						− 0.07	.001	0.22	− 0.31	.754
Subjective anxiety	0.22	.007	0.22	0.99	.322						0.17	.004	0.23	0.74	.456
* R*^2^	.03					.06					0.07				
Preoccupation with thinness regressed on:															
Physical concerns						− 0.11	.004	0.09	− 1.21	.225	− 0.15	.007	0.09	− 1.62	.105
Cognitive concerns						0.22	.012	0.11	1.97	.049	0.18	.007	0.11	1.60	.109
Social concerns						**0.25**	**.015**	**0.11**	**2.16**	**.031**	0.20	.009	0.12	1.69	.090
Somatic symptoms	− 0.24	.008	0.17	− 1.43	.152						− 0.28	.011	0.17	− 1.67	.095
Subjective anxiety	**0.56**	**.045**	**0.17**	**3.32**	**.001**						**0.45**	**.026**	**0.18**	**2.55**	**.011**
* R*^2^	.12					.13					.16				

Bolded entries indicate significant results at or below the .05 level. *sr*^2^ = squared semipartial correlations, calculated using the supplemental rsquareCalc() function from Hayes [95]. Reduced models were specified according to the extra DV approach described in Hayes [95]. These models were estimated using WLSMV estimation in lavaan

In addition to this full model, we estimated two reduced models: (1) a model that only included the two BAI factors as regressors (*x*-variables); and (2) a model that only included the three ASI-3 factors as regressors (*x*-variables);. The rationale for including these reduced models was to afford easy calculation of the *R*-squared change ($\Delta {R}^{2}$), and to facilitate comparison of whether and how results might shift when partialling out shared variance from the other constructs. To afford accurate calculation of $\Delta {R}^{2}$ between models, we estimated both reduced models using the extra DV approach described in Hayes [95].^9^

#### Latent variable regression results

Standardized results from our latent variable regression models are displayed in Table 3, fit using WLSMV estimation in lavaan. Squared semipartial correlations (*sr*^2^ values) were calculated using the supplemental rsquareCalc()function from Hayes [95]. Table 4 presents all pairwise model-implied correlations between our final model regressors and outcomes in the model.

**Table 4:** Model-implied regressor-outcome correlations

	1	2	3	4	5	6	7	8	9	10
1. ASI: physical concerns										
2. ASI: cognitive concerns	0.79									
3. ASI: social concerns	0.79	0.84								
4. BAI: somatic symptoms	0.63	0.65	0.64							
5. BAI: subjective anxiety	0.65	0.67	0.66	0.93						
6. Binging	0.26	0.32	0.20	0.23	0.30					
7. Purging	0.25	0.31	0.12	0.09	0.17	0.55				
8. Diet/carb restriction	− 0.05	− 0.02	− 0.05	− 0.03	− 0.02	0.15	0.29			
9. Pressure to eat	0.06	0.19	0.17	0.15	0.17	− 0.11	0.32	0.15		
10. Preoccupation with thinness	0.26	0.34	0.35	0.28	0.34	0.60	0.40	0.45	0.03	

Correlations were estimated using the what = “cor.all” argument in the lavInspect() function in lavaan, applied to the final latent variable regression model

As Table 3 shows, the AS subscales exhibit significant regression relationships with both the binging and purging outcomes, with or without controlling for general anxiety. Whereas higher levels of cognitive concerns were associated with higher latent propensities to binge,^10^ the reverse appeared to be true of social concerns, such that higher degrees of social concerns were associated with lower propensities to binge. Comparing the model *R*^2^ from the full model to that of reduced model 1, the set of AS factors uniquely accounted for $\Delta {R}^{2}$ = 0.17 – 0.11 = 0.06, or 6% of the variance in binging propensity.

Furthermore, higher levels of cognitive concerns and lower levels of social concerns were significantly associated with higher purging propensities, controlling for somatic symptoms and subjective anxiety. The squared semipartial correlations associated with these relationships are even more pronounced than for the binging outcome (see Table 3): cognitive concerns uniquely accounted for 10.5% of the variance in latent binging propensities whereas social concerns uniquely accounted for 7.8% of the variance in latent binging propensities, controlling for each other, and for all other model regressors (*x*-variables);. Comparing the model *R*^2^ for the purging outcome to that of reduced model 1, adding the AS factors results in $\Delta {R}^{2}$ = 0.22 – 0.06 = 0.16, suggesting that AS accounts for 16% of the variance in latent binging propensity, controlling for the factors of general anxiety.

These results suggest that AS exerts significant associations with binging and purging outcomes, above and beyond general anxiety. Specifically, higher levels of cognitive concerns are positively associated with the propensities to binge and purge whereas higher levels of social concerns are negatively associated with these outcomes, possibly suggesting a protective or buffering influence. The AS subscales were not associated with the remaining eating outcomes in this sample–although, neither were the BAI subscales, except the subjective anxiety and panic subscale, which was associated with higher levels of latent preoccupation with thinness when controlling for all other regressors.

Table 5 presents the results of our final model, after re-estimating it in Mplus using Bayesian estimation to fit the model and handle missing data. The pattern of results is comparable in direction, (relative) strength, and (Bayesian analogues to) ‘significance.’ These results only serve to strengthen our confidence in the robustness of our main model findings to different approaches to missing data handling.

**Table 5:** Standardized latent variable regression results using Bayesian estimation in Mplus

	Est	Posterior *SD*	*p*	95% Credible interval		Sig
				Lower bound	Upper bound	
Binging regressed on:						
Physical concerns	0.03	0.13	.418	− 0.24	0.28	
Cognitive concerns	0.52	0.18	.001	0.18	0.90	*
Social concerns	− 0.42	0.19	.013	− 0.80	− 0.06	*
Somatic symptoms	− 0.77	0.29	.003	− 1.34	− 0.21	*
Subjective anxiety	0.98	0.30	< .001	0.39	1.58	*
Purging regressed on:						
Physical concerns	0.13	0.20	.262	− 0.27	0.51	
Cognitive concerns	0.83	0.29	.001	0.31	1.46	*
Social concerns	− 0.61	0.32	.011	− 1.41	− 0.08	*
Somatic symptoms	− 0.91	0.40	.009	− 1.8	− 0.13	*
Subjective anxiety	0.84	0.42	.019	0.04	1.74	*
Diet foods/carb reduction regressed on:						
Physical concerns	− 0.06	0.11	.294	− 0.27	0.16	
Cognitive concerns	0.12	0.14	.209	− 0.16	0.41	
Social concerns	− 0.10	0.15	.249	− 0.40	0.18	
Somatic symptoms	− 0.08	0.22	.357	− 0.55	0.34	
Subjective anxiety	0.11	0.23	.315	− 0.32	0.60	
Pressure to eat regressed on:						
Physical concerns	− 0.21	0.12	.036	− 0.44	0.02	
Cognitive concerns	0.26	0.14	.031	− 0.01	0.56	
Social concerns	0.12	0.15	.221	− 0.19	0.42	
Somatic symptoms	− 0.06	0.22	.392	− 0.49	0.36	
Subjective anxiety	0.12	0.23	.299	− 0.31	0.56	
Desire for thinness regressed on:						
Physical concerns	− 0.10	0.09	.149	− 0.29	0.09	
Cognitive concerns	0.27	0.12	.011	0.04	0.50	*
Social concerns	0.03	0.13	.387	− 0.22	0.28	
Somatic symptoms	− 0.46	0.20	.005	− 0.88	− 0.09	*
Subjective anxiety	0.65	0.21	< .001	0.27	1.08	*

*Indicates parameter estimates whose 95% posterior credible interval does not contain zero

## Discussion

In the present study, we found that in a large sample of young adults, AS cognitive and social dimensions were associated with ED symptomatology, and most importantly, that these associations went beyond those of general anxiety. It is important to mention that most research to date has treated AS as a sole construct, without analyzing the differential contributions of the three AS dimensions. The present study expands our understanding of the unique and distinctive contributions of each AS dimension (beyond the influences of general anxiety) associated with EDs outcomes. We motivate future researchers to unravel these specific associations and further prove/establish AS risk and resilience effects in the development and maintenance of EDs.

Our results suggested that the cognitive dimension of AS behaved as a risk pathway for eating pathology, while the social dimension revealed a possible resilience or protective effect for EDs. (i.e., specifically for binging and purging outcomes). This suggests that individuals who exhibit heightened fears regarding their anxiety-related thoughts, such as catastrophic interpretations of physical sensations, may be more prone to engaging in binge eating and purging. This positive association has important practice implications, as treatments should address the specific anxiety-related thoughts, and help reframe the catastrophic interpretations of physical sensations, to develop healthier coping mechanisms and reduce reliance on these maladaptive eating behaviors. On the other hand, individuals more concerned about their anxiety symptoms being publicly observable and more preoccupied about negative evaluations from others, may be at lower risk for engaging in binging and purging behaviors. Again, these findings are relevant for clinical practice and the development of new interventions that could target these increased concerns about social evaluations. Our results support previous findings looking at the associations between AS and binging, suggesting higher AS levels associated with greater calorie consumption [40], greater eating expectancies or feeling out of control [56, 96], or binging behaviors [55]. Literature on the associations among AS and purging is scarce.

On the other hand, the non-significant results across diet and carb restriction, pressure to eat and preoccupation with thinness could also be a result of the non-clinical sample selected. In fact, in a clinical sample, drive for thinness was significantly associated with AS [97].

Due to the high comorbidity among anxiety and eating pathologies, our results on the association of AS with ED beyond the highly comorbid generalized anxiety have clinical potential for differential and transdiagnostic prevention and intervention for EDs. By accounting for general anxiety we were able to focus specifically on the association of AS with EDs outcomes, proving a more targeted and unique understanding of these associations. This has clinical implications, allowing practitioners to better understand the complex interplay among factors involved in eating pathologies, and most importantly, to disentangle the high comorbidity among anxiety and EDs, to help develop more effective prevention and treatment interventions.

This study also adds to the emerging body of literature questioning the traditional factor structure of the EAT-26 in non-SWAG stereotyped samples [71, 72, 75]. The uniqueness of our sample (i.e., mostly female and Hispanic), led to establish a new four-factor model of the EAT-26, similar to previous studies [98, 99]. Our findings reaffirm the need to embrace diversity in ED research, as proposed by Halbeisen et al. [69] and the need to critically assess widely used ED assessment tools (i.e., EAT-26) that may perform differently across diverse understudied samples. Further, the rising rates of EDs across these largely neglected populations- of different race/identity, gender, and sexual identity groups [100, 101], older adults [102], and diverse socio-economic statuses [103, 104]—stresses the need for greater diversity in future research of the etiology and symptomatic expression and to develop diversity-affirming and culturally-sensitive assessment tools for EDs.

The current study has important limitations. First, it is based on a convenience sample recruited from the university population and composed of over 70% females, over 60% Hispanic, and a reported average EAT-26 total score below the clinical cutoff. This makes it difficult to generalize our findings to other genders, cultures, diverse demographic and educational backgrounds, or clinical populations. Future studies should consider including a broader spectrum of severity in EDs. Second, the cross-sectional nature of this study prevents us from establishing causal inferences between AS subscales and ED outcomes. Future research should examine through longitudinal designs how AS dimensions predict and impact in a temporal manner EDs risk or resilience. It is also important to note that the current dataset was collected in 2018, thus the reported levels and experience of anxiety and eating behaviors might have aggravated in the current time as a result of the health pandemic in 2020 [105]. One final methodological limitation is our reliance on single item indicators for certain constructs in our model. Naturally, it would be more ideal to have multiple indicators for assessing binging and purging behaviors, as exemplified by our approach in estimating latent factors for the other subscales. Nonetheless, using the single binging item, for example, allowed us to assess binging behaviors rather than omitting this construct from the model or attempting to force this binging item to hang together with other scale items that do not share the same meaning (that is, that do not ask about binging behavior directly). That is, the decision to incorporate this single item was based upon both statistical considerations in terms of model fit and EFA results, as well as substantive concerns in terms of the meaning of the items in the EAT-26 questionnaire. From our standpoint, the available options were limited to either incorporating single item indicators as standalone outcomes, a choice substantively congruent with our rationale, or alternatively, omitting such items altogether, thereby missing any opportunity to assess the (semipartial) associations of anxiety sensitivity and general anxiety with these constructs within our model. In addition, previous studies have introduced new factorial structures for the EAT-26 including single item indicators [74, 106]. Ultimately, it is important to be aware that our reliance on single item indicators may have introduced measurement error and reduced the precision of our estimates in these parts of the model.

In addition to demonstrating the differential pathways of association of AS with disordered eating beyond generalized anxiety, our model provides therapeutic potential to reduce the risk of EDs. Emerging and young adulthood has been associated with elevated levels of stress and anxiety [107] that can impact AS and disordered eating. It has been argued that AS is a critical indicator and transdiagnostic treatment target for EDs [53, 54]. Thus, our model has important implications for practice, as it demonstrates associations between AS and ED pathology and suggests that both of these associated factors need to be considered when assessing risk. In fact, Fletcher et al. [108] argued that effective EDs interventions are dependent on addressing comorbid non-eating behaviors such as anxiety.

In conclusion, our results suggest that individuals high in AS may rely on maladaptive eating (i.e., binging and purging) in an effort to regulate the experience of AS. Our differential results regarding the AS subscales underscore not only the complexity of eating pathologies, but also the importance of considering the unique fears and concerns associated with AS in the assessment and treatment of EDs. Anxiety sensitivity should be targeted in transdiagnostic treatment approaches to reduce risk and develop effective prevention and intervention strategies for EDs.

### Supplementary Information

**Additional file 1**. Supplemental Materials.

## Data Availability

The data that support the findings of this study are available from the corresponding author upon reasonable request.

## Footnotes

1 The lifetime comorbidity among anxiety disorders and anorexia nervosa (AN) was 47.9%, 80.6% for bulimia nervosa (BN), and 65.1% for binge eating disorder (BED).

2 Although we note that in the case of the EAT-26, this is only after recoding all items as instructed in the scale’s scoring instructions.

3 Note that because the majority of fit indices suggested acceptable fit of the BAI and because we hoped to test our incremental validity hypotheses controlling for the BAI as it is conventionally conceptualized– that is, we did not wish to reimagine the factor structure of the BAI in order to improve model fit and thereby leave our analyses open to potentially justifiable criticisms that we were not truly controlling for general anxiety, as envisaged by Beck, but were, rather, controlling for some other, perhaps related, construct(s) derived from the same items–we decided to retain the two-factor BAI in our final model, rather than attempting to improve the fit using post-hoc model modification strategies.

4 Note that the resulting factor structure, described below, remained near-identical when we relaxed this cutoff value to standardized loadings of .6.

5 These analyses were conducted by the quantitative methodologist working on the project, [NAME REDACTED FOR BLIND REVIEW], who was not thoroughly acquainted with the background literature on the EAT-26 measure at the time of the initial analyses.

6 However, in addition to fitting this CFA model to the dataset used in the present study, we also cross-validated the 4-factor structure of the EAT in three additional (validation) datasets collected by the same lab. The 4-factor version of the eat displayed in Table-2 resulted in good model fit in all three validation samples. See the Additional file 1 for further details.

7 Note that, as stated, Fig. 2 depicts a *path diagram* of a latent variable *regression* model; not a *causal diagram*. As such, using standard path diagrammatic notation, the one-headed arrows in this diagram represent regression coefficients–no more, no less. Although we theorize that general anxiety and anxiety sensitivity can act as antecedents to disordered eating (but, as noted above, the past literature suggests that they might also, at times, act as consequents), we fully recognize the inability of cross-sectional data to speak to temporal precedence, let alone causality. Because the one-headed arrows here represent hypothesized *associations*, not ironclad *causes*, one peer reviewer thoughtfully suggested that we change all one-headed arrows in the diagram to two-headed arrows. Though we appreciate the judicious and careful approach implied by this suggestion, we leave the diagram as-is, including the one-headed arrows in order to accurately depict the latent variable regressions as run in Mplus. Indeed, it is this regression-based specification that allowed us to test our incremental validity hypothesis by partialling out overlapping variance between the AS and BAI factors on the *x*-side of the model. Of course, the effect sizes of greatest interest in these analyses are the *squared semipartial correlations* and *R*-squared change values reported below, which can be interpreted as measures of (semi)partial *association* without recourse to causal language. Thus, ultimately, we fully trust our readers to understand the assumptions and caveats that come hand-in-hand with any regression analyses conducted on cross-sectional data, including latent variable regression analyses such as this one.

8 Note that due to convergence errors when running our full multifactor model, we had to drop one of the two purging items from our full SEM analyses. These two items were highly correlated (polychoric *r* = .82) and overlapping in their meaning, bordering on collinear. Thus, we opted to retain item 25, “have the impulse to vomit after meals,” as an observed outcome in place of the two-indicator purging factor described in the previous section.

9 Note that both reduced models, by design, returned identical model fit to our full SEM model. Therefore, these redundant fit statistics are omitted from Table 1.

10 This interpretation is in line with the latent response formulation for binary and ordinal outcomes.

## References

[1] Galmiche M, Déchelotte P, Lambert G, Tavolacci MP. Prevalence of eating disorders over the 2000–2018 period: A systematic literature review. In: American journal of clinical nutrition, vol. 109. Oxford: Oxford University Press; 2019. p. 1402–13. 10.1093/ajcn/nqy342 31051507

[2] Brewerton TD (2007). Eating disorders, trauma, and comorbidity: focus on PTSD. Eat Disord.

[3] Hudson JI, Hiripi E, Pope HG, Kessler RC (2007). The prevalence and correlates of eating disorders in the national comorbidity survey replication. Biol Psychiatry.

[4] Hughes EK (2012). Comorbid depression and anxiety in childhood and adolescent anorexia nervosa: prevalence and implications for outcome. Clin Psychol.

[5] Goel NJ, Sadeh-Sharvit S, Trockel M, Flatt RE, Fitzsimmons-Craft EE, Balantekin KN (2021). Depression and anxiety mediate the relationship between insomnia and eating disorders in college women. J Am Coll Health.

[6] Garcia SC, Mikhail ME, Keel PK, Burt SA, Neale MC, Boker S (2020). Increased rates of eating disorders and their symptoms in women with major depressive disorder and anxiety disorders. Int J Eat Disord.

[7] Godart NT, Flament MF, Perdereau F, Jeammet P (2002). Comorbidity between eating disorders and anxiety disorders: a review. Int J Eat Disord.

[8] Konstantellou A, Campbell M, Eisler I, Simic M, Treasure J (2011). Testing a cognitive model of generalized anxiety disorder in the eating disorders. J Anxiety Disord..

[9] Pallister E, Waller G (2008). Anxiety in the eating disorders: understanding the overlap. Clin Psychol Rev.

[10] Swinbourne JM, Touyz SW (2007). The co-morbidity of eating disorders and anxiety disorders: a review. Eur Eat Disord Rev.

[11] Zerwas S, Von HA, Watson H, Gottfredson N, Bulik CM (2014). Childhood anxiety trajectories and adolescent disordered eating: findings from the NICHD study of early child care and youth development. Int J Eat Disord.

[12] Bandelow B, Michaelis S. Epidemiology of anxiety disorders in the 21st century [Internet]. 2015. Available from: www.dialogues-cns.org 10.31887/DCNS.2015.17.3/bbandelow PMC4610617 26487813

[13] Ori APS, Wieling M, van Loo HM (2023). Longitudinal analyses of depression, anxiety, and suicidal ideation highlight greater prevalence in the northern Dutch population during the COVID-19 lockdowns. J Affect Disord.

[14] Hart LM, Granillo MT, Jorm AF, Paxton SJ. Unmet need for treatment in the eating disorders: a systematic review of eating disorder specific treatment seeking among community cases. Vol. 31, Clinical Psychology Review. 2011. p. 727–35. 10.1016/j.cpr.2011.03.004 21501580

[15] Kuntz L. A life and death measure: Eating disorder treatment. Psychiatric Times. 2021.

[16] Kessler RC, Merikangas KR (2004). The National Comorbidity Survey Replication (NCS-R): background and aims. Int J Methods Psychiatr Res.

[17] Berg KC, Crosby RD, Cao L, Peterson CB, Engel SG, Mitchell JE (2013). Facets of negative affect prior to and following binge-only, purge-only, and binge/purge events in women with bulimia nervosa. J Abnorm Psychol.

[18] Lavender JM, De Young KP, Wonderlich SA, Crosby RD, Engel SG, Mitchell JE (2013). Daily patterns of anxiety in anorexia nervosa: Associations with eating disorder behaviors in the natural environment. J Abnorm Psychol.

[19] Haynos AF, Roberto CA, Attia E (2015). Examining the associations between emotion regulation difficulties, anxiety, and eating disorder severity among inpatients with anorexia nervosa. Compr Psychiatry.

[20] Touchette E, Henegar A, Godart NT, Pryor L, Falissard B, Tremblay RE (2011). Subclinical eating disorders and their comorbidity with mood and anxiety disorders in adolescent girls. Psychiatry Res.

[21] Hussenoeder FS, Conrad I, Engel C, Zachariae S, Zeynalova S, Glaesmer H (2021). Analyzing the link between anxiety and eating behavior as a potential pathway to eating-related health outcomes. Sci Rep.

[22] Catone G, Pisano S, Muzzo G, Corrado G, Russo K, Maiorano A (2020). A glance into psychiatric comorbidity in adolescents with anorexia nervosa. Minerva Pediatr.

[23] Rojo-Moreno L, Arribas P, Plumed J, Gimeno N, García-Blanco A, Vaz-Leal F (2015). Prevalence and comorbidity of eating disorders among a community sample of adolescents: 2-year follow-up. Psychiatry Res..

[24] Swanson SA, Crow SJ, Le Grange D, Swendsen J, Merikangas KR (2011). Prevalence and correlates of eating disorders in adolescents: results from the national comorbidity survey replication adolescent supplement. Arch Gen Psychiatry.

[25] Arbués ER, Abadía BM, López JMG, Serrano EE, García BP, Vela RJ (2019). Eating behavior and its relationship with stress, anxiety, depression, and insomnia in university students. Nutr Hosp.

[26] Kaye WH, Bulik CM, Thornton L, Barbarich N, Masters K (2004). Comorbidity of anxiety disorders with anorexia and bulimia nervosa. Am J Psychiatry.

[27] Sassaroli S, Bertelli S, Decoppi M, Crosina M, Milos G, Ruggiero GM (2005). Worry and eating disorders: a psychopathological association. Eat Behav.

[28] Levinson CA, Byrne M (2015). The fear of food measure: a novel measure for use in exposure therapy for eating disorders. Int J Eat Disord.

[29] Levinson CA, Brosof LC, Ram SS, Pruitt A, Russell S, Lenze EJ (2019). Obsessions are strongly related to eating disorder symptoms in anorexia nervosa and atypical anorexia nervosa. Eat Behav.

[30] Sternheim L, Startup H, Saeidi S, Morgan J, Hugo P, Russell A (2012). Understanding catastrophic worry in eating disorders: process and content characteristics. J Behav Ther Exp Psychiatry.

[31] Berkman ND, Lohr KN, Bulik CM. Outcomes of eating disorders: A systematic review of the literature. Vol. 40, International Journal of Eating Disorders. 2007. p. 293–309. 10.1002/eat.20369 17370291

[32] Swinbourne J, Hunt C, Abbott M, Russell J, St Clare T, Touyz S (2012). The comorbidity between eating disorders and anxiety disorders: prevalence in an eating disorder sample and anxiety disorder sample. Aust N Z J Psychiatry.

[33] Ranta K, Väänänen J, Fröjd S, Isomaa R, Kaltiala-Heino R, Marttunen M (2017). Social phobia, depression and eating disorders during middle adolescence: longitudinal associations and treatment seeking. Nord J Psychiatry.

[34] Taylor S (1995). Anxiety sensitivity: theoretical perspectives and recent findings. Behav Res Ther.

[35] Anderson ER, Hope DA (2009). The relationship among social phobia, objective and perceived physiological reactivity, and anxiety sensitivity in an adolescent population. J Anxiety Disord.

[36] Walsh TM, Stewart SH, McLaughlin E, Comeau N (2004). Gender differences in Childhood Anxiety Sensitivity Index (CASI) dimensions. J Anxiety Disord.

[37] Frazier LD, Waid LD (1999). Influences on anxiety in later life: the role of health status, health perceptions, and health locus of control. Aging Mental Health.

[38] Reiss S, Peterson RA, Gursky DM, McNally RJ (1986). Anxiety sensitivity, anxiety frequency and the prediction of fearfulness. Behav Res Ther.

[39] Reiss S. Expectancy model of fear, anxiety, and panic. Vol. 11, Clinical Psychology Rmew. 1991.

[40] Hearon BA, Quatromoni PA, Mascoop JL, Otto MW (2014). The role of anxiety sensitivity in daily physical activity and eating behavior. Eat Behav.

[41] McNally RJ (2002). Anxiety sensitivity and panic disorder. Biol Psychiatry.

[42] Paulus DJ, Gallagher MW, Bartlett BA, Tran J, Vujanovic AA (2018). The unique and interactive effects of anxiety sensitivity and emotion dysregulation in relation to posttraumatic stress, depressive, and anxiety symptoms among trauma-exposed firefighters. Compr Psychiatry.

[43] Barlow DH (1991). The nature of anxiety: anxiety, depression, and emotional disorders. Chronic anxiety: generalized anxiety disorder and mixed anxiety-depression.

[44] Taylor S (2020). Anxiety sensitivity. Clinical handbook of fear and anxiety: Maintenance processes and treatment mechanisms.

[45] Ehlers A (1995). A 1-year prospective study of panic attacks: clinical course and factors associated with maintenance. J Abnorm Psychol.

[46] Otto MW, Pollack MH, Fava M, Uccello R, Rosenbaum JF (1995). Elevated Anxiety Sensitivity Index scores in patients with major depression: correlates and changes with antidepressant treatment. J Anxiety Disord.

[47] Silverman WK, Goedhart AW, Barrett P, Turner C (2003). The facets of anxiety sensitivity represented in the Childhood Anxiety Sensitivity Index: Confirmatory analyses of factor models from past studies. J Abnorm Psychol.

[48] Viana AG, Kiel EJ, Alfano CA, Dixon LJ, Palmer CA (2017). The contribution of temperamental and cognitive factors to childhood anxiety disorder symptoms: a closer look at negative affect, behavioral inhibition, and anxiety sensitivity. J Child Fam Stud.

[49] Craske MG (2012). Transdiagnostic treatment for anxiety and depression. Depress Anxiety.

[50] Ellard KK, Fairholme CP, Boisseau CL, Farchione TJ, Barlow DH (2010). Unified protocol for the transdiagnostic treatment of emotional disorders: protocol development and initial outcome data. Cogn Behav Pract.

[51] Stice E. Risk and maintenance factors for eating pathology: A meta-analytic review. In: Psychological bulletin, vol. 128. Washington: American Psychological Association Inc; 2002. p. 825–48. 10.1037/0033-2909.128.5.825 12206196

[52] Wildes JE, Marcus MD (2011). Development of emotion acceptance behavior therapy for anorexia nervosa: a case series. Int J Eat Disord.

[53] Fulton JJ, Lavender JM, Tull MT, Klein AS, Muehlenkamp JJ, Gratz KL (2012). The relationship between anxiety sensitivity and disordered eating: the mediating role of experiential avoidance. Eat Behav.

[54] Smits JAJ, Otto MW, Powers MB, Baird SO (2019). Anxiety sensitivity as a transdiagnostic treatment target. The clinician’s guide to anxiety sensitivity treatment and assessment.

[55] DeBoer LB, Tart CD, Presnell KE, Powers MB, Baldwin AS, Smits JAJ (2012). Physical activity as a moderator of the association between anxiety sensitivity and binge eating. Eat Behav.

[56] Hearon BA, Utschig AC, Smits JAJ, Moshier SJ, Otto MW (2013). The role of anxiety sensitivity and eating expectancy in maladaptive eating behavior. cognit ther res.

[57] Anestis MD, Selby EA, Fink EL, Joiner TE (2007). The multifaceted role of distress tolerance in dysregulated eating behaviors. Int J Eat Disord.

[58] Taylor S, Zvolensky MJ, Cox BJ, Deacon B, Heimberg RG, Ledley DR (2007). Robust dimensions of anxiety sensitivity: development and initial validation of the Anxiety Sensitivity Index-3. Psychol Assess.

[59] Garner DM, Olmsted MP, Bohr Y, Garfinkel PE (1982). The Eating Attitudes Test: psychometric features and clinical correlates. Psychol Med.

[60] Peterson RA, Reiss S (1992). Anxiety sensitivity index manual.

[61] Espel-Huynh HM, Muratore AF, Virzi N, Brooks G, Zandberg LJ (2019). Mediating role of experiential avoidance in the relationship between anxiety sensitivity and eating disorder psychopathology: A clinical replication. Eat Behav.

[62] Fink E, Bodell L, Smith A, Joiner T (2013). The joint influence of disordered eating and anxiety sensitivity on the acquired capability for suicide. Cognit Ther Res.

[63] Westfall J, Yarkoni T (2016). Statistically controlling for confounding constructs is harder than you think. PLoS ONE.

[64] Beck AT, Epstein N, Brown G, Steer RA (1988). An inventory for measuring clinical anxiety: psychometric properties. J Consult Clin Psychol.

[65] Mintz LB, O’Halloran MS (2000). The eating attitudes test: validation with DSM-IV eating disorder criteria. J Pers Assess.

[66] Rivas T, Bersabé R, Jiménez M, Berrocal C (2010). The eating attitudes test (EAT-26): reliability and validity in spanish female samples. Spanish J Psychol.

[67] Papini NM, Jung M, Cook A, Lopez NV, Ptomey LT, Herrmann SD, Kang M (2022). Psychometric properties of the 26-item eating attitudes test (EAT-26): an application of rasch analysis. J Eat Disord.

[68] Desai MN, Miller WC, Staples B, Bravender T (2008). Risk factors associated with overweight and obesity in college students. J Am Coll Health.

[69] Halbeisen G, Brandt G, Paslakis G (2022). A plea for diversity in eating disorders research. Front Psychiatry.

[70] Khaled SM, Kimmel L, Le Trung K (2018). Assessing the factor structure and measurement invariance of the eating attitude test (EAT-26) across language and BMI in young Arab women. J Eat Disord.

[71] Rogoza R, Brytek-Matera A, Garner DM (2016). Analysis of the EAT-26 in a non-clinical sample. Arch Psychiatry Psychother.

[72] Spivak-Lavi Z, Peleg O, Tzischinsky O, Stein D, Latzer Y (2021). Differences in the factor structure of the eating attitude test-26 (Eat-26) in different cultures in Israel: Jews, muslims, and christians. Nutrients.

[73] Belon KE, Smith JE, Bryan AD, Lash DN, Winn JL, Gianini LM (2011). Measurement invariance of the Eating Attitudes Test-26 in Caucasian and Hispanic women. Eat Behav.

[74] Rutt CD, Coleman KJ (2005). Examining the relationships among built environment, physical activity, and body mass index in El Paso. TX Prev Med.

[75] Schaefer LM, Anderson LM, Simone M, O’Connor SM, Zickgraf H, Anderson DA (2019). Gender-based differential item functioning in measures of eating pathology. Int J Eat Disord.

[76] Cole DA, Preacher KJ (2014). Manifest variable path analysis: Potentially serious and misleading consequences due to uncorrected measurement error. Psychol Methods.

[77] Rosseel Y (2012). lavaan: an R package for structural equation modeling. J Stat Softw..

[78] R Core Team. R: A language and environment for statistical computing [Internet]. Vienna, Austria: R Foundation for Statistical Computing; 2021. Available from: http://r-project.org/

[79] Muthén LK, Muthén B (2017). Mplus user’s guide.

[80] Taylor S, Zvolensky MJ, Cox BJ, Deacon B, Heimberg RG, Ledley DR (2022). Robust dimensions of anxiety sensitivity: development and initial validation of the Anxiety Sensitivity Index-3. Psychol Assess.

[81] Finney SJ, DiStefano C, Hancock GR, Mueller RO (2013). Nonnormal and categorical data in structural equation modeling. Structural equation modeling: a second course.

[82] Rhemtulla M, Brosseau-Liard PÉ, Savalei V (2012). When can categorical variables be treated as continuous? A comparison of robust continuous and categorical SEM estimation methods under suboptimal conditions. Psychol Methods.

[83] Muthén B (2004). Mplus technical appendices.

[84] Enders CK, Keller BT. Blimp User’s Manual (Version 3) [Internet]. Los Angeles, CA; 2021. Available from: www.appliedmissingdata.com/multilevel-imputation.html

[85] Hu L, Bentler PM (1999). Cutoff criteria for fit indexes in covariance structure analysis: conventional criteria versus new alternatives. Struct Equ Model.

[86] Ocker LB, Lam ETC, Jensen BE, Zhang JJ (2007). Psychometric properties of the eating attitudes test. Meas Phys Educ Exerc Sci..

[87] Maïano C, Morin AJS, Lanfranchi MC, Therme P (2013). The Eating Attitudes Test-26 revisited using exploratory structural equation modeling. J Abnorm Child Psychol..

[88] Papini NM, Jung M, Cook A, Lopez NV, Ptomey LT, Herrmann SD (2022). Psychometric properties of the 26-item Eating Attitudes Test (EAT-26): an application of Rasch analysis. J Eat Disord..

[89] Kline RB (2016). Principles and practice of structural equation modeling.

[90] Bollen KA (1989). Structural equations with latent variables.

[91] Kenny DA, Milan S, Hoyle RH (2012). Identification: A non-technical discussion of a technical issue. Handbook of structural equation modeling.

[92] Kenny DA, Kashy DA, Bolger N, Gilbert D, Fiske S, Lindzey G (1998). Data analysis in social psychology. The handbook of social psychology.

[93] Thurstone LL (1935). The vectors of mind: Multiple-factor analysis for the isolation of primary traits.

[94] Thurstone LL (1947). Multiple-factor analysis.

[95] Hayes T (2021). R-squared change in structural equation models with latent variables and missing data. Behav Res Methods.

[96] Kauffman BY, Shepherd JM, Bakhshaie J, Zvolensky MJ (2021). Anxiety sensitivity in relation to eating expectancies among college students. J Am Coll Health.

[97] Anestis MD, Holm-Denoma JM, Gordon KH, Schmidt NB, Joiner TE (2008). The role of anxiety sensitivity in eating pathology. Cognit Ther Res.

[98] Maïano C, Morin AJS, Lanfranchi MC, Therme P (2013). The Eating Attitudes Test-26 revisited using exploratory structural equation modeling. J Abnorm Child Psychol.

[99] Ocker LB, Lam ETC, Jensen BE, Zhang JJ. Psychometric properties of the eating attitudes test. Vol. 11, Measurement in physical education and exercise science. 2007.

[100] Coelho JS, Suen J, Clark BA, Marshall SK, Geller J, Lam PY. Eating Disorder Diagnoses and Symptom Presentation in Transgender Youth: a Scoping Review. Vol. 21, Current Psychiatry Reports. Current Medicine Group LLC 1; 2019. 10.1007/s11920-019-1097-x 31617014

[101] Sangha S, Oliffe JL, Kelly MT, McCuaig F. Eating disorders in males: how primary care providers can improve recognition, diagnosis, and treatment. In: American journal of men’s health, vol. 13. Thousand Oaks: SAGE Publications Inc.; 2019. p. 1–12. 10.1177/1557988319857424 PMC6560809 31184292

[102] Mangweth-Matzek B, Hoek HW. Epidemiology and treatment of eating disorders in men and women of middle and older age. In: Current opinion in psychiatry, vol. 30. Williams and Wilkins: Lippincott; 2017. p. 446–51. 10.1097/YCO.0000000000000356 PMC5690315 28825955

[103] Qian J, Wu Y, Liu F, Zhu Y, Jin H, Zhang H, et al. An update on the prevalence of eating disorders in the general population: a systematic review and meta-analysis. In: Eating and weight disorders, vol. 27. Deutschland: Springer Science and Business Media; 2022. p. 415–28. 10.1007/s40519-021-01162-z PMC8933366 33834377

[104] Santomauro DF, Melen S, Mitchison D, Vos T, Whiteford H, Ferrari AJ (2021). The hidden burden of eating disorders: an extension of estimates from the Global Burden of Disease Study 2019. Lancet Psychiatry.

[105] Arcelus J, Mitchell AJ, Wales J, Nielsen S (2011). Mortality rates in patients with anorexia nervosa and other eating disorders: a meta-analysis of 36 studies. Arch Gen Psychiatry.

[106] Swarnameenaa G, Durairaj J, Madhavan VK, Hariharan N, Arunachaleeswaran P, Venkatraman L (2023). The Tamil version of Eating Attitudes Test-26: reliability and factor structure among persons with schizophrenia. Indian J Psychiatry.

[107] Potterton R, Austin A, Flynn M, Allen K, Lawrence V, Mountford V (2021). “I’m truly free from my eating disorder”: emerging adults’ experiences of FREED, an early intervention service model and care pathway for eating disorders. J Eat Disord.

[108] Fletcher BC, Kupshik GA, Uprichard S, Shah S, Nash AS (2008). Eating disorders and concurrent psychopathology: a reconceptualisation of clinical need through Rasch analysis. Eur Eat Disord Rev.

