# Supplementary material for: Beyond generalized anxiety: the association of anxiety sensitivity with disordered eating
Source: J Eat Disord. 2023 Oct 2;11:173. doi: 10.1186/s40337-023-00890-0 (PMC10544544; doi:10.1186/s40337-023-00890-0)
Supplement: Supplementary file 1 — Additional file 1. Supplemental Materials. [file 40337_2023_890_MOESM1_ESM.docx]

**Online Supplemental Materials**

In addition to the EFA and CFA analyses reported in the main manuscript, we cross-validated the fit of our proposed factor structure for the EAT (described in the main text and shown in Table 2 in the main paper) using three additional datasets. The three validation datasets represented data from three different studies collected by the same lab that happened to have included data on the EAT-26 measure. The original aims and sample characteristics of these three validation datasets are provided below. Two of the datasets were collected using undergraduate students who participated via the psychology subject pool at Florida International University (FIU). The third dataset was collected on members of the U.S. military.

**Validation Datasets**

***Health Behaviors Survey 2016***

This study aimed to examine health-related and eating behaviors in college students. The sample consisted of 482 college students (77% female) between the ages of 18–53 (*M* = 21.35, *SD* = 3.83). Participants represented a diverse group of [cultures](https://www.sciencedirect.com/topics/medicine-and-dentistry/culture): 37.6% were Cuban, 20.7% were South American, 8.2% were Central American, 4% were Dominican, 3.6% were Puerto Rican, and 1.8% were Mexican. The remaining 24.1% self-identified as belonging to more than one cultural group. These ethnic demographics align closely to those within the greater metropolitan area where the study was conducted ([Miami-Dade County Planning Research, 2011](https://www.sciencedirect.com/science/article/pii/S1471015317301265#bb0150)). [Body Mass Index](https://www.sciencedirect.com/topics/medicine-and-dentistry/body-mass-index) (BMI) was calculated by self-reported height and weight (kg/m^2^). The mean BMI for the sample was 24.66 (SD = 4.93), with 60.4% of the sample reporting a normal BMI (between 18.5 and 24.9), 25.5% reporting an overweight BMI (between 25 and 29.9), 11.8% reporting an obese BMI (> 30), and 2.3% reporting an underweight BMI (< 18.5), based on the World Health Organization (WHO) classification system ([World Health Organization, 2000](https://www.sciencedirect.com/science/article/pii/S1471015317301265#bb0285)).

***Health Literacy Survey 2012***

This study aimed to examine health-related behaviors in college students. The data are part of a large, cross-sectional data set on college student health behaviors. Given the study’s aims of extending prior research on non-Hispanic white samples to a Hispanic sample, the sample was restricted to participants self-identifying as either Hispanic or non-Hispanic white. Participants included 1339 college students (64.1 % female) attending a large, South Florida university and ranging in age from 18 to 35 years (M = 21, SD = 3.22). The sample was predominantly Hispanic (82%, N = 1048), and of the Hispanic participants, 35 % were Cuban, 17.4 % were South American, 7.4 % were Central American, 3.6 % were Puerto Rican, 3.3 % were Dominican, 1.2 % were Mexican, and .6 % were Spanish, making this sample different from most prior studies on eating disorder risk in predominantly Mexican-American Hispanic populations. These sample characteristics are representative of the university and greater South Florida demographics.

***Military Sexual Trauma Sample***

This study aimed to examine linkages among military sexual trauma and eating pathology in currently enlisted and veteran military personnel. Participants (N = 123) of this study identified as male (59.3%) and female (40.7%), were primarily between the ages of 25 and 49 years old (range = 18-65+), and self-reported belonging to the following racial/ethnic backgrounds: Black/African American (14.6%), Asian/Asian Pacific Islander (15.4%), Native American/Alaskan Native (8.1%), Native Hawaiian or other pacific islander (1.6%), White, non-Hispanic (47.2%), Other (5.7%), Prefer not to say (1.6%), Prefer to self-describe (4.9%). There were also several military branches represented in this sample: Coast Guard (4.1%), Marines (13.0%), Navy (24.4%), Army (45.5%), Air Force 8.9%, and Other (1.6%). The majority of participants were veterans (44.7%), but a significant number were serving on active duty (28.5%), while the remaining reported serving in the reserve (13.0%) and the national guard (13.8%). Finally, most participants were high ranking enlisted (43.1%), between the ranks of E5-E9. About 35% were low ranking enlisted, between ranks E1-E-4, 4.1% were warrant officers, 9.8% junior officers, 4.1% senior officers, and finally 1.6% were attending officer preparatory school.  2.5% listed their rank as unknown.

**Supplemental Results and Discussion**

Online supplemental Table O1 presents the fit statistics returned from (A) the primary dataset reported in our main paper (the *training* dataset, on which we build our 4-factor model) and (B) the three *validation datasets* that we used to assess the fit of our proposed factor structure. All CFAs were conducted using the same methods described in the main paper (e.g., WLSMV estimation for categorical indicators). Examining Table O1, it is easy to see that fit is very good across nearly all fit statistics in all datasets: all CFI and TLI values were > .95, all RMSEA values were > .08, and all SRMR values were ≥ .08. Additionally, model chi-square tests were non-significant for all validation datasets except the Health Literacy 2012 dataset, which featured the largest sample size (*N* = 1676). Given the well-known sensitivity of the model chi-square test to sample size, this result is neither especially troubling nor especially surprising. Given the excellent fit suggested by the remaining statistics (CFI, TLI, RSMEA, and SRMR), all of which (with the arguable exception of the SRMR) make explicit adjustments to alleviate the chi-square’s sample size dependence, we still feel that the fit of our CFA to this dataset is very strong and, overall, very encouraging.

The strong fit of our CFA model in the three validation datasets reassures us that the model’s good fit to our primary dataset does not appear to be an artifact of *overfitting* a single (training) dataset. This also lends support to our guiding principles of considering both the original EFA results *and* the substantive meaning of the items grouped by the EFA when deciding which items to retain for each factor in our final model. Recall that our initial EFA, presented in the main paper Table 2, grouped the EAT-26 items “Feel extremely guilty after eating” and “Think about burning up calories when I exercise” with three items measuring *preoccupation with thinness*. Although grouping these items with the remaining *preoccupation with thinness* items could be entertained as plausible and these items did, indeed, appear to group with the other *preoccupation* items in the training dataset, scrutiny of their substantive meaning suggested that these items did not align as closely in their meaning to the other three items: “Am terrified of being overweight”, “Am preoccupied with a desire to be thinner”, and “Am preoccupied with the thought of having fat on my body.”

Under the statistical assumptions of the common factor model, it seems quite reasonable to assume that participants higher in their levels of a latent, true score “preoccupation with thinness” factor would necessarily endorse higher responses to all three of these latter items, without exception. By contrast, considering the statistical assumptions of the common factor model, it seemed *plausible* that participants higher in their levels of a latent, true score “preoccupation with thinness” factor *might* endorse higher responses to both “Feel extremely guilty after eating” and “Think about burning up calories when I exercise” (and, reiterating, the high loadings for these items in main paper Table 2 suggest that participants in our sample, in fact, did), but we reasoned that this outcome did not necessarily seem guaranteed in future samples. For example, if a future sample of participants who are preoccupied with their thinness are not also exercisers, they might be unlikely to endorse item 12 despite exhibiting higher levels of this latent factor. Similarly, if participants in a future sample were not overeaters and/or were lower in neuroticism around food (e.g., the type of individuals who experience little guilt when adhering to a strict regimen of eating only light salads every day), these individuals may be less prone to endorsing item 10 despite being high in their true levels of preoccupation with thinness. Because of these concerns, and because a latent variable requires only 2-3 indicators to reach identification in a larger SEM, the inclusion of these two lower-loading indicators was not required in our final model and we opted to ultimately drop them from further analyses in the main paper. The strong fit of our confirmatory factor models in the three validation sets shown here suggests that this strategy of retaining only items that exhibited both statistical *and* substantive evidence of strong grouping may, indeed, have been a prudent one.

**Table O1**

| *Fit Statistics from CFAs of the EAT-26 Factor Structure from Main Paper Table 2 Conducted on (A) the Original Training Dataset (from the Main Paper) and (B) Three Validation Datasets* | | | | |
| --- | --- | --- | --- | --- |
|  | Health Behaviors Survey 2018 (Training Data) | Health Behaviors Survey 2016 | Health Literacy Survey 2012 | Risk and Resilience in Military Personnel |
| *N* | 786 | 934 | 1676 | 140 |
| Chi-square | 132.13 | 151.63 | 299.12 | 42.56 |
| *df* | 38 | 38 | 38 | 38 |
| *p* | < .001 | < .001 | < .001 | 0.281 |
| CFI | 0.97 | 0.98 | 0.97 | 1.00 |
| TLI | 0.96 | 0.97 | 0.96 | 0.99 |
| RMSEA | 0.06 | 0.06 | 0.06 | 0.03 |
| 90% CI | [0.05, 0.07] | [0.05, 0.07] | [0.06, 0.07] | [0.00, 0.07] |
| *p_close_* | 0.156 | 0.117 | < .001 | 0.769 |
| SRMR | 0.09 | 0.08 | 0.05 | 0.07 |
| Note: The Health Behaviors Survey 2018 dataset is dataset reported in the body of the main paper. This acted as the *training data* on which we ran our primary EFA and initial CFA analyses and is included here for the sake of direct comparison. The *test datasets* used to cross-validate our proposed factor structure are shown in the rightmost three columns. | | | | |
